# Supplementary material for: Manipulating Steady Heat Conduction by Sensu-shaped Thermal Metamaterials
Source: Sci Rep. 2015 May 14;5:10242. doi: 10.1038/srep10242 (PMC4431466; doi:10.1038/srep10242)
Supplement: Supplementary Information [file srep10242-s1.doc]

**Supplementary Information**

**Manipulating Steady Heat Conduction by *Sensu*-shaped Thermal Metamaterials**

Tiancheng Han^1*^, Xue Bai^2,3,4^, Dan Liu^2,3,4^, Dongliang Gao^2^, Baowen Li^3,4,5^, John T. L. Thong^2,4^, and Cheng-Wei Qiu^2,4#^

^1^School of Physical Science and Technology, Southwest University, Chongqing 400715, China

^2^Department of Electrical and Computer Engineering, National University of Singapore, 4 Engineering Drive 3, Republic of Singapore

^3^Department of Physics and Centre for Computational Science and Engineering, National University of Singapore, Singapore 117546, Republic of Singapore

^4^NUS Graduate School for Integrative Sciences and Engineering, National University of Singapore, Kent Ridge 119620, Republic of Singapore

^5^Center for Phononics and Thermal Energy Science, School of Physics Science and Engineering, Tongji University, 200092, Shanghai, China

Corresponding authors: [*tchan123@swu.edu.cn](mailto:*tchan123@swu.edu.cn); [#](mailto:#eleqc@nus.edu.sg)eleqc@nus.edu.sg

1. **Simulation for the creation of a uniform heating region by using SSTM or copper**

For quantitative comparison, the temperature of the center is marked out. We can see that the temperature of the center in Fig. S1(a) is higher than that in Fig. S1(b) 14.2 K. Clearly, the uniform heating performance of Fig. S1(a) (with SSTM) is much better than that in Fig. S1(b) (with bare copper).


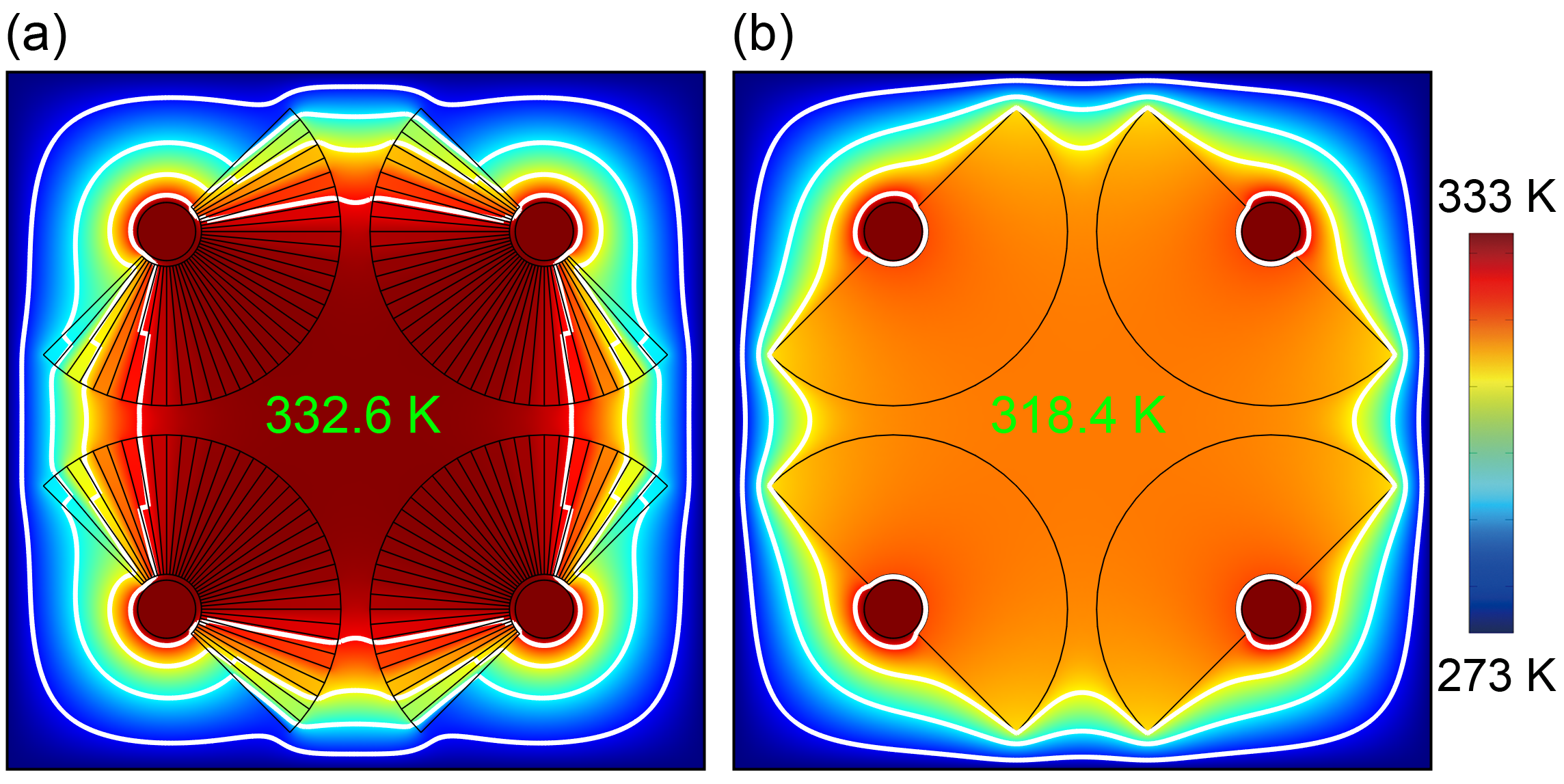


**Figure S1.** The temperature distributions of four distant point-sources respectively enclosed by SSTM or copper. (a) With SSTM. (b) With bare copper. For quantitative comparison, the temperature of the center is marked out. Isothermal lines are shown in white.

1. **The performance of Fig. 2(a) with different distance between two adjacent SSTM units**


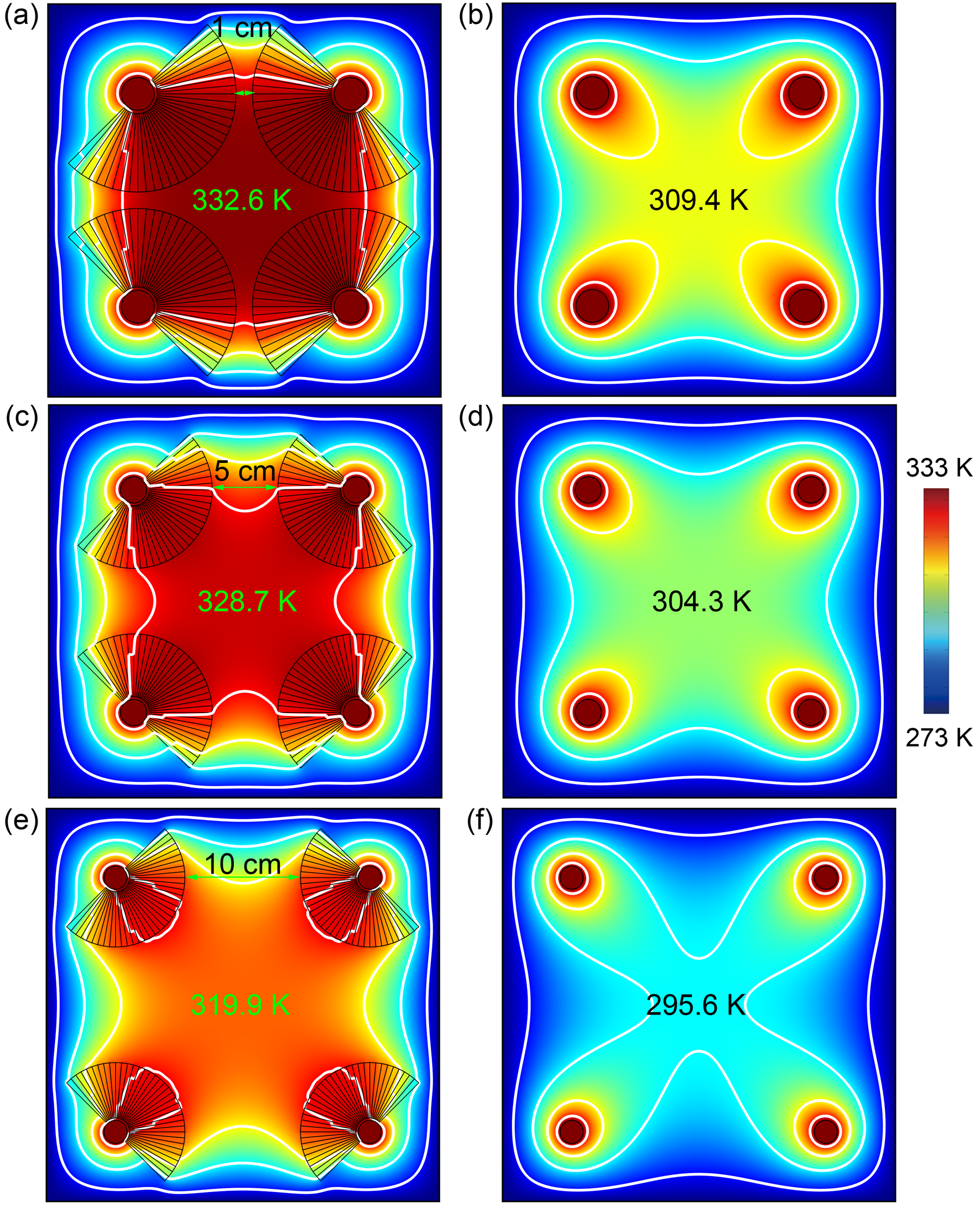


**Figure S2.** Performance of Fig. 2(a) with different distance between two adjacent SSTM units. Left column corresponds to simulated thermal profile of four distant heat-sources separately enclosed by four SSTM units when (a) *d*=1 cm, (c) *d*=5 cm, and (e) *d*=10 cm, respectively. Right column corresponds to simulated thermal profile of four distant heat-sources without SSTM when (b) *d*=1 cm, (d) *d*=5 cm, and (f) *d*=10 cm, respectively. For quantitative comparison, the temperature of the center is marked out. Isothermal lines are shown in white.

The performance is degraded with the increase of *d*. However, the temperature distribution for the case with SSTM (*d*=10 cm in Fig. S2(e)) is still much higher than the case without SSTM (*d*=1cm in Fig. S2(b)).

1. **Thermal focusing effect with copper wedges, copper quadrants, or SSTM**


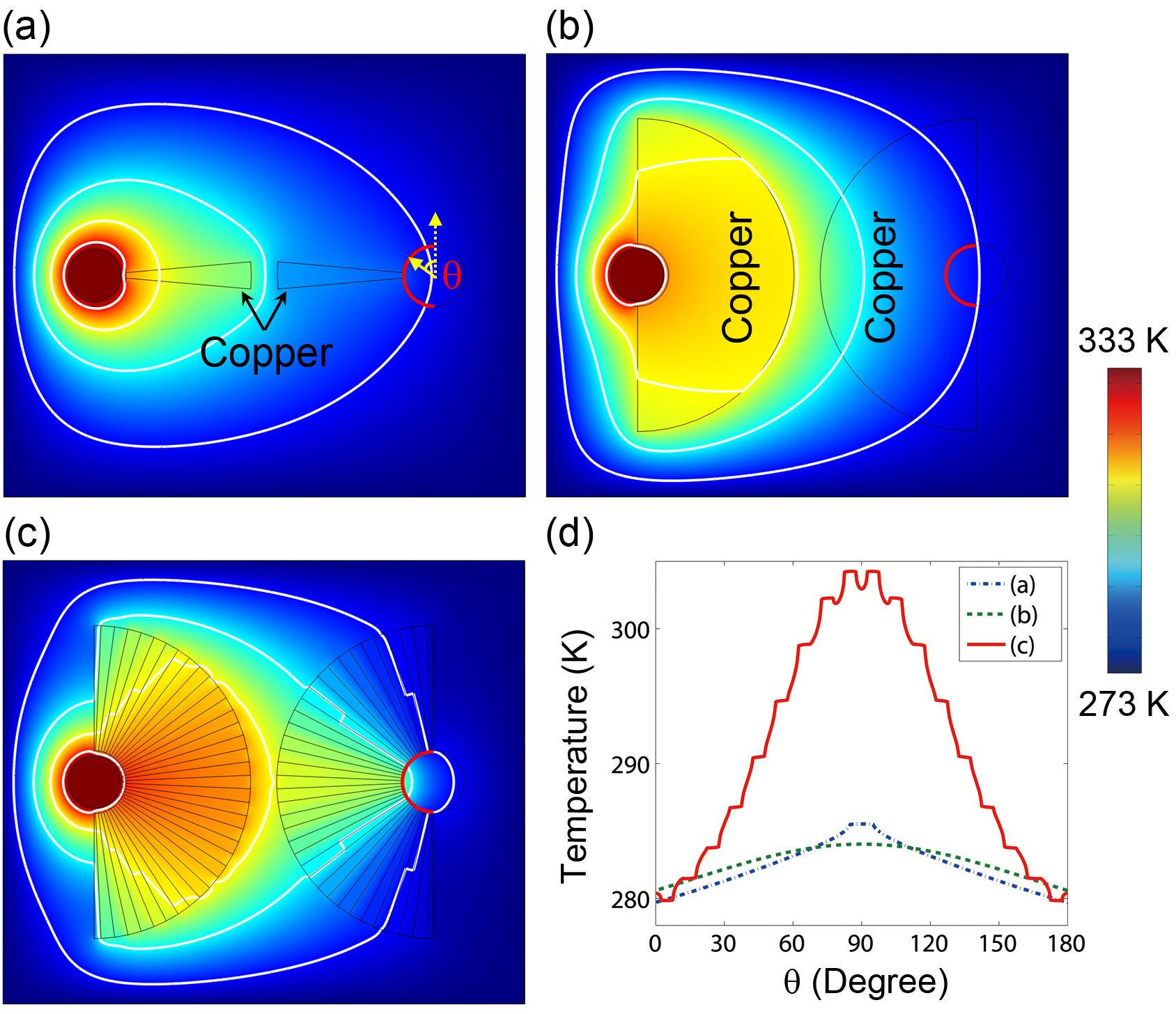


**Figure S3.** The thermal focusing effect with copper wedges, copper quadrants, or SSTM. (a) With copper wedges. (b) With copper quadrant. (c) With SSTM. (d) Quantitative contrast of temperature distribution along semicircular red line. Isothermal lines are shown in white.

1. **Investigation of concentration effect with bare copper or PDMS**


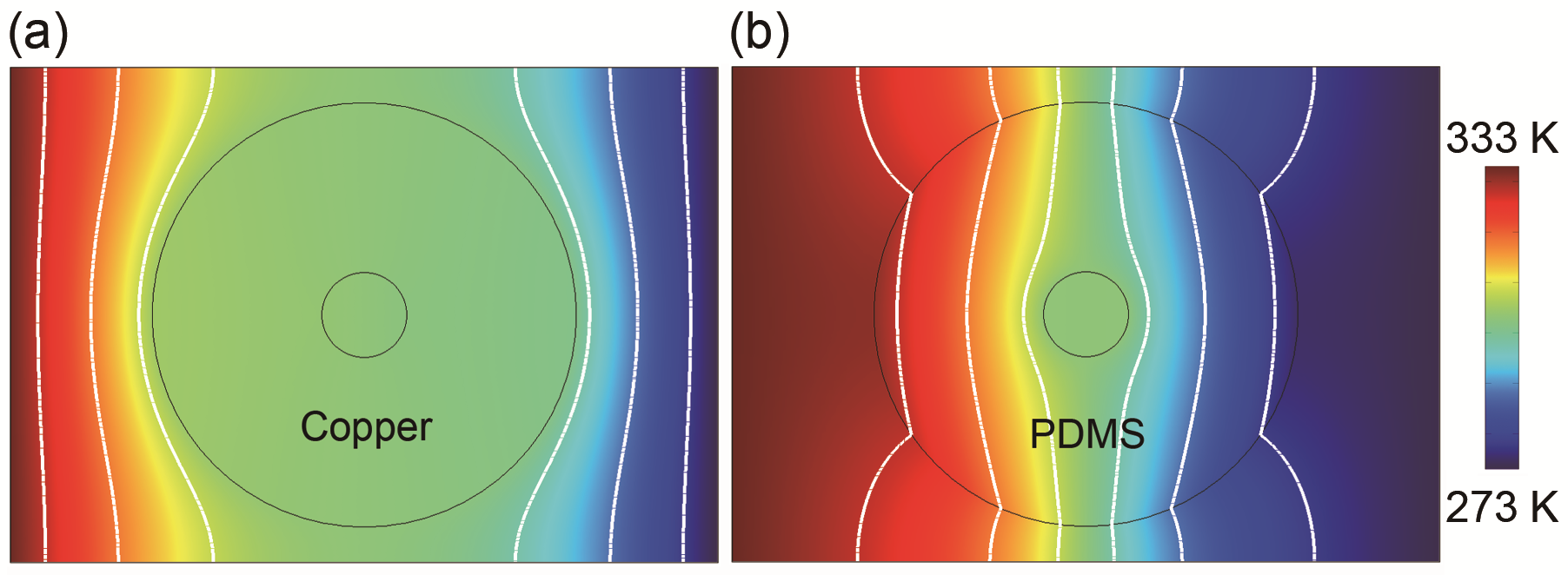


**Figure S4.** No concentration effect with bare copper or PDMS. (a) With bare copper. (b) With bare PDMS. Isothermal lines are shown in white.

1. **Simulation results of manipulating *dc* currents by using SSTM**

In the simulation setup, we use the same geometrical parameters and material components as those in the thermal regime. We choose *a*=1.2 cm and *b*=6 cm. An SSTM unit is composed of 18 copper wedges and 18 PDMS wedges, which is embedded in stainless steel. The electrical conductivities of copper and stainless steel are $\sigma_{Cu}=5.9\times10^{7}$ S/m and $\sigma_{b}=1.3\times10^{6}$ S/m, respectively. PDMS can be regarded as ideal insulating material. Figure S5 shows the simulation results of manipulating *dc* currents by using SSTM, which demonstrates bifunctional property of the proposed SSTM.


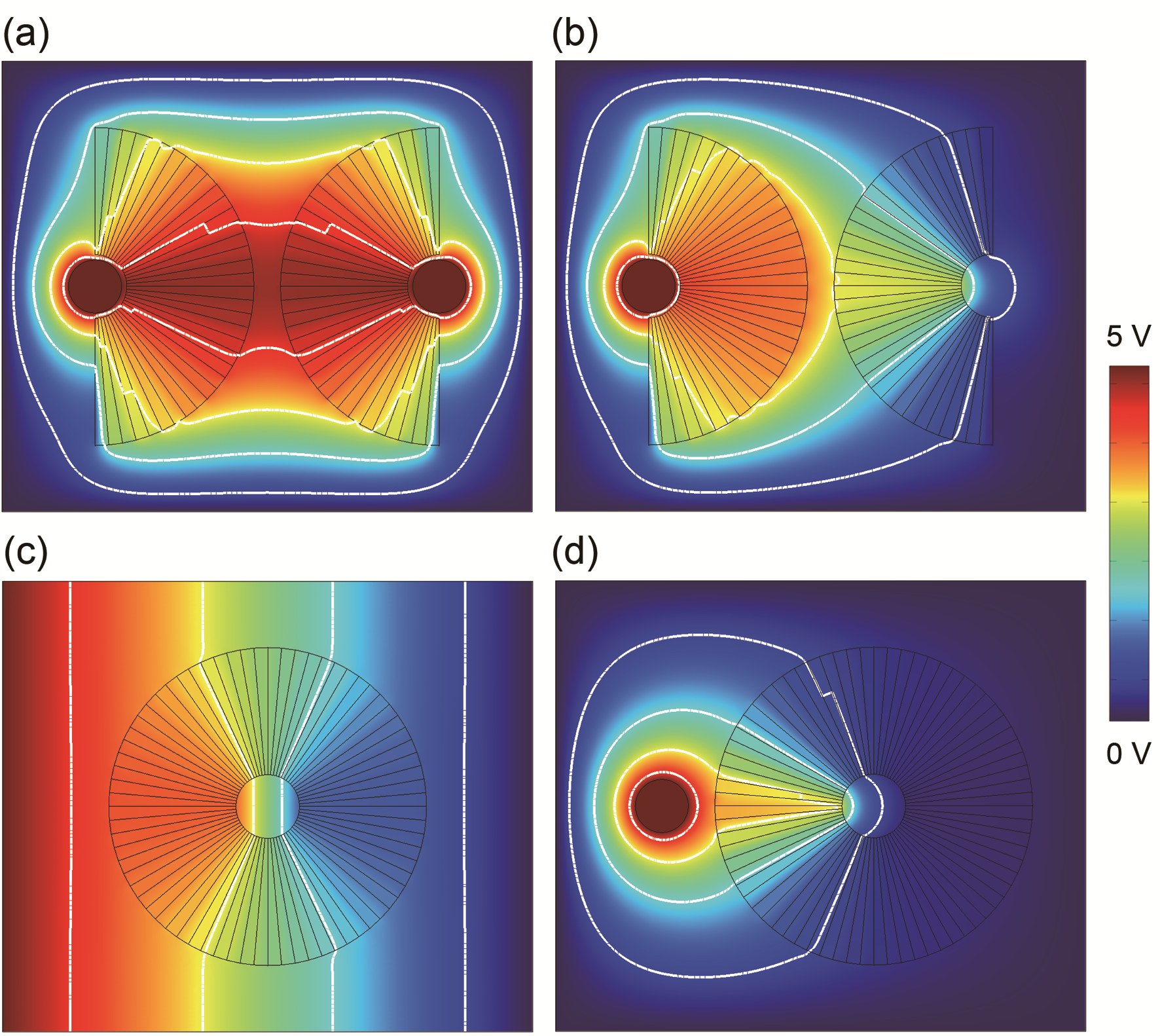


**Figure S5.** Simulation results of manipulating *dc* currents by using SSTM. (a) Forming a nearly constant voltage region between two cylindrical *dc*-sources. (b) Demonstration of *dc* focusing. Demonstration of *dc* concentration in uniform *dc* field (c) and non-uniform *dc* field (d). Equipotential lines are shown in white.
